# Untargeted metabolomics reveals divergent metabolic profiles between the predatory *Arma chinensis* and the Phytophagous *Halyomorpha halys*

**DOI:** 10.1093/jisesa/ieag005

**Published:** 2026-02-02

**Authors:** Zhihan Su, Wenyan Xu, Luyao Fu, Dianyu Liu, Changjin Lin, Xiaoyu Yan, Yu Chen, Yichen Wang, Xiaolin Dong, Chenxi Liu

**Affiliations:** College of Agriculture, Yangtze University, Jingzhou, China; Sino-American Biological Control Laboratory, Institute of Plant Protection, Chinese Academy of Agricultural Sciences, Beijing, China; Sino-American Biological Control Laboratory, Institute of Plant Protection, Chinese Academy of Agricultural Sciences, Beijing, China; Sino-American Biological Control Laboratory, Institute of Plant Protection, Chinese Academy of Agricultural Sciences, Beijing, China; College of Agriculture, Yangtze University, Jingzhou, China; Sino-American Biological Control Laboratory, Institute of Plant Protection, Chinese Academy of Agricultural Sciences, Beijing, China; Sino-American Biological Control Laboratory, Institute of Plant Protection, Chinese Academy of Agricultural Sciences, Beijing, China; College of Agriculture, Yangtze University, Jingzhou, China; Sino-American Biological Control Laboratory, Institute of Plant Protection, Chinese Academy of Agricultural Sciences, Beijing, China; College of Agriculture, Yangtze University, Jingzhou, China; Sino-American Biological Control Laboratory, Institute of Plant Protection, Chinese Academy of Agricultural Sciences, Beijing, China; Sino-American Biological Control Laboratory, Institute of Plant Protection, Chinese Academy of Agricultural Sciences, Beijing, China; College of Agriculture, Yangtze University, Jingzhou, China; Sino-American Biological Control Laboratory, Institute of Plant Protection, Chinese Academy of Agricultural Sciences, Beijing, China

**Keywords:** *Arma chinensis*, *Halyomorpha halys*, untargeted metabolomics, feeding habit divergence

## Abstract

*Arma chinensis* (Fallou) (a predatory insect) and *Halyomorpha halys* (Stål) (a phytophagous insect) exhibit distinct feeding ecologies. This contrast provides a model system to investigate metabolic divergence in insects, which remains insufficiently characterized. To address this, we employed untargeted metabolomics for comparing the global metabolic profiles of these 2 species. Significant differences were detected between *A. chinensis* and *H. halys*, with 194 and 195 differentially abundant metabolites identified in females and males, respectively. The metabolic profile of *A. chinensis* was characterized by an enrichment of lipids and lipid-like molecules. In contrast, *H. halys* exhibited an enrichment of organic acids, their derivatives, and plant-derived secondary metabolites, consistent with its phytophagous diet. Sex-specific metabolic patterns were also observed: females showed higher lipid accumulation, a pattern often associated with reproductive investment in insects, whereas males displayed a relative increase in metabolites related to protein synthesis. This study elucidates the distinct metabolomic signatures associated with different feeding habits for 2 closely related insect species. These findings provide a foundation for further investigation into the physiological correlates of dietary ecology and may inform future research into pest management strategies.

## Introduction

Insects, the most abundant animal group on Earth, exhibit remarkable diversity, largely due to variations in their feeding habits, which range from strict phytophagy and carnivory (predation or parasitism) to omnivory. These divergences drive adaptive evolution in morphology, physiology, and behavior, influencing ecological interaction networks. Understanding the physiological and metabolic mechanisms underlying dietary shifts or specialization constitutes a central topic in insect evolutionary biology and physiological ecology. Over 75% of insect species utilize plant resources (phytophagy); however, diversification into alternative nutritional strategies, such as predation, can promote niche partitioning (Forister et al. 2012).

*Arma chinensis* (Fallou) belongs to the family Pentatomidae and order Hemiptera. It is primarily distributed across East Asia, including China, Mongolia, the Korean Peninsula, and Japan (Fu et al. 2024). *Halyomorpha halys* (Stål), another member of the Pentatomidae family (Hemiptera), is native to China, Japan, and Korea (Mele et al. 2022). Phylogenetic studies based on gene sequencing (Sparks et al. 2020) and morphological classification (Zou et al. 2012) have revealed a close evolutionary relationship between *A. chinensis* and *H. halys.* Despite this genetic proximity, these species have developed divergent feeding habits (Cheng et al. 2025), making them an ideal model system for investigating feeding habit-driven metabolic adaptations among closely related taxa.

*Arma chinensis* is a hemimetabolous insect, progressing through 3 main developmental stages: egg, nymph (with 5 instars) (Fu et al. 2024), and adult. It effectively suppresses agricultural and forestry pests within the Lepidoptera and Coleoptera orders (Zhang et al. 2017). Its high consumption capacity, robust predatory ability, and notable adaptability to varied environments have established *A. chinensis* as a highly valued agent in biological pest control, often referred to as a “field guardian” in agricultural settings (Shen et al. 2018). In contrast, *H. halys* is a typical phytophagous pest with a broad host range. Using piercing–sucking mouthparts, *H. halys* penetrates plant tissues—including fruits and young shoots—to extract sap, causing fruit deformity, abscission, and shoot withering. This causes considerable economic losses to fruit trees, such as pears, apples, and peaches, as well as certain vegetables and soybeans (Lee et al. 2013, Leskey and Nielsen 2018, Ferrari et al. 2025).

Despite their potential habitat overlap (eg orchard ecosystems), their core niches are markedly divergent. *Arma chinensis* occupies the predator trophic level, indirectly benefiting plants by regulating populations of phytophagous insects, including *H. halys* nymphs. Conversely, *H. halys* has a direct negative impact on primary producers (plants), which establishes a potential predator–prey relationship and competition for resources, such as overwintering sites.

Predatory insects like *A. chinensis* primarily derive energy from the lipids and proteins of their prey (Wilder et al. 2013). Lipids are vital for supporting essential behavioral and physiological functions (Jensen et al. 2011), while proteins serve as both energy sources and substrates for enzyme synthesis and tissue development (Walter et al. 2017). Efficient utilization of lipid- and protein-rich diets is thus essential for predatory insects. In contrast, phytophagous insects such as *H. halys* must adapt to the limited nutrient content of plant sap and to diverse plant secondary metabolites, including phenolics and alkaloids (Smilanich et al. 2016).

Previous research on these pentatomids has largely focused on their morphology, bionomics, and ecological behavior (such as predatory activity in *A. chinensis*, host selection, and damage caused by *H. halys*), as well as the development of effective control technologies—particularly those targeting *H. halys*. However, our understanding of how dietary divergence shapes the intrinsic metabolic phenotypes of closely related species at the physiological level remains limited.

Metabolic profile differences have also been reported between sexes, often linked to variations in reproductive investment, such as vitellogenesis in females and testicular development in males, as well as differences in energy allocation strategies, hormone levels, and behavior. For example, sexually biased gene expression related to sugar metabolism has been observed in the gut of male *Drosophila melanogaster* (Hudry et al. 2019), along with sex-specific metabolite patterns in *Caenorhabditis elegans* (Burkhardt et al. 2023), sex-based differences in environmental sensitivity (Teder and Kaasik 2023), and diet-modulated immune function differences between sexes in *Bactrocera tryoni* (Fanson et al. 2013). However, the regulatory role of sexual dimorphism in metabolic adaptation remains unclear. This underscores the importance of considering sex as a dimension of any metabolomic comparison between *A. chinensis* and *H. halys*.

Metabolomics provides a comprehensive and unbiased methodological framework for characterizing the global metabolic state of organisms under specific conditions. Rapid advancement in this field has highlighted the roles of metabolism and small-molecule metabolites in diverse biological processes (Wishart 2019), including insect cold adaptation (Hayward and Colinet 2023), viral infection responses (Wang et al. 2024b), metabolic regulation (Li et al. 2023b), and drought tolerance (Ryabova et al. 2020). Untargeted metabolomics—using high-resolution mass spectrometry and chromatographic separation—enables the detection of metabolites without a priori selection, making it particularly suitable for exploratory research and biomarker discovery (Mayack et al. 2020). Multivariate analysis methods, including principal component analysis (PCA), partial least squares-discriminant analysis (PLS-DA), and orthogonal PLS-DA (OPLS-DA), are commonly used to differentiate metabolic profiles among experimental groups and identify key differential metabolites and enriched metabolic pathways involved in underlying biological mechanisms.

Previous studies have demonstrated that feeding habits significantly shape insect metabolic profiles. Diet-induced metabolic alterations have been observed in *Bombyx mori* (Li et al. 2023a), while diverse food sources (Oliveira et al. 2022) and growth regulator stress conditions (Liu et al. 2023) influence the metabolic profiles of *Spodoptera frugiperda* and *Helicoverpa armigera*, respectively. These findings highlight the impact of feeding habits on insect metabolic networks and provide insights into trophic cascades, host–microbiota interactions, and pest control strategies.

This study employed untargeted metabolomics and a controlled experimental design (considering species and sex) to compare the global metabolic profiles of *A. chinensis* and *H. halys*. The primary objectives were to evaluate the overall differences in metabolic profiles between predatory and phytophagous pentatomids, identify key species- and sex-specific metabolites, analyze core metabolic pathways and their biological relevance, and explore how these metabolic differences reflect adaptive evolutionary strategies regarding energy acquisition, nutrient utilization, detoxification, and reproductive investment. The findings are expected to contribute to our understanding of dietary adaptation mechanisms in insects and provide theoretical support and potential metabolic biomarkers for mass-rearing and efficacy optimization of *A. chinensis* for biological control and for discovering novel targets for the green management of *H. halys*.

## Materials and Methods

### Instruments

The following instruments were used in this study: a refrigerated centrifuge (Model 5430 R, Eppendorf, Hamburg, Germany), a water purification system (Model Labtower EDI, Thermo Scientific, Germering, Germany), a high-throughput tissue grinder (Model TL-48R, Jingxin, Shanghai, China), an ultrasonic cleaner (Model SB-800D, SCIENTZ, Ningbo, China), and a vacuum concentrator (Model SCIENTZ-1LS, SCIENTZ, Ningbo, China). Metabolite separation was performed using an ultra-high performance liquid chromatography (UPLC) system (Vanquish Flex, Thermo Scientific) coupled to a high-resolution Orbitrap mass spectrometer (Orbitrap Exploris 120, Thermo Scientific) for detection.

### Experimental Insects

The initial *A. chinensis* population was collected from a field in Beijing, China. Successive generations were reared in the laboratory on an artificial prey diet containing *Antheraea pernyi* pupae until a stable colony was established. Larvae were housed in rearing containers (8.5 cm diameter × 10 cm height) with mesh lids. Absorbent cotton moistened with water was formed into balls and placed on top of the mesh lid to supply drinking water to *A. chinensis*. Fresh *A. pernyi* pupae were provided every 3 days as food. The rearing containers were maintained in a climate-controlled incubator set at 26 ± 1 °C, 65% ± 5% relative humidity, and a 16 L:8D photoperiod. *Antheraea pernyi* pupae used to feed *A. chinensis* were stored at 4 °C to preserve their condition.

Adult *H. halys* individuals were collected from Sanming City, Fujian Province, China. An experimental colony was established and maintained in a rearing container (32 × 20 × 18 cm). Fresh corn was provided as the food source, while moistened absorbent cotton balls supplied water. Food was replaced, moisture was replenished, and eggs were removed every 3 days. The rearing containers were maintained in a climate-controlled incubator at 26 ± 1 °C, 65% ± 5% relative humidity, and a 16 L:8D photoperiod. Corn used for feeding *H. halys* was stored at 4 °C to maintain its freshness.

Newly emerged adult females and males of *A. chinensis* and *H. halys* were selected for analysis. Before experimentation, all specimens were acclimated under uniform laboratory incubator conditions (26 ± 1 °C, 65% ± 5% relative humidity, and 16 L:8D photoperiod) for 2 wk.

### Metabolomic Sequencing

Newly emerged adults of both species, after 2 wk of acclimation, were collected and divided into 4 groups: (i) *A. chinensis* females (AF), (ii) *A. chinensis* males (AM), (iii) *H. halys* females (HF), and (iv) *H. halys* males (HM). The samples were surface-cleaned with 75% ethanol, rinsed twice with distilled water, flash-frozen in liquid nitrogen, and stored at −80 °C until analysis. Metabolomic sequencing was performed by Shanghai Personalbio Technology Co., Ltd. Whole-insect homogenates were used in this study; 6 biological replicates per group were included, selected based on project time constraints and the complexity of obtaining insect species within a specific seasonal window. Moreover, this sample size is widely accepted and commonly applied in untargeted metabolomics comparisons, providing robust and reliable data (Zhang et al. 2024b).

Metabolite extraction was performed as follows. Thirty milligrams of the sample was weighed into a 2 ml centrifuge tube, followed by the addition of 200 μl of pre-cooled water and 2 steel beads. The mixture was homogenized using a high-throughput tissue grinder at 55 Hz for 60 s, and the grinding step was repeated once. Thereafter, 800 μl of methanol:acetonitrile (1:1, v/v) was added, and the tube was ultrasonicated for 30 min. Afterward, the sample was frozen at −20 °C for 45 min and subsequently centrifuged at 15,294 *×* *g* and 4 °C for 20 min. The supernatant (800 μl) was collected and concentrated to dryness under vacuum. The residue was reconstituted in 150 μl of 50% methanol containing 5 ppm 2-chlorophenylalanine by vortexing for 30 s, followed by centrifugation at 15,294 *×* *g* at 4 °C for 10 min. The supernatant was filtered through a 0.22 μm membrane, and the filtrate was transferred into a vial. For quality control (QC), 10 to 20 μl of each sample filtrate was pooled to prepare a QC sample, which was used to evaluate the instrument stability and data reliability.

### Chromatography-Mass Spectrometry Analysis

The chromatographic conditions were as follows.

The ACQUITY UPLC HSS T3 (100 Å, 1.8 μm, 2.1 × 100 mm) column was used, with a column temperature of 40 °C, autosampler temperature of 8 °C, and injection volume of 2 μl. The flow rate was set at 0.4 ml/min. The mobile phase consisted of 0.1% formic acid in water (A) and acetonitrile (containing 0.1% formic acid; B).

The Thermo Orbitrap Exploris 120 mass spectrometer was used to collect DDA mass spectrometric data in the positive and negative ion modes using Xcalibur software (version 4.7; Thermo). For Heated Elektrospray lonization source (HESI)source, the following settings were used: spray voltage: 3.5/−3.0 kV; sheath gas: 40 arb; auxiliary gas: 15 arb; capillary temperature: 325 °C; auxiliary gas temperature: 300 °C; primary resolution: 60,000; scan range: 100 to 1,000 m/z, AGC target: Standard; and Max IT: 100 ms. The top 4 ions were screened for secondary fragmentation using the following parameters: dynamic exclusion time: 8 s; secondary resolution: 15,000; HCD collision energy: 30%; AGC target: Standard; and Max IT: Auto.

All formal and QC samples were loaded following the chromatography and mass spectrometry methods described previously. Before running the main samples, QC samples were injected 2 to 4 times to balance the system. Throughout the process, a QC sample was run every 5 to 10 samples to support ongoing data evaluation and maintain QC.

### Metabolite Data Processing and Identification

The raw format data were imported into the commercial software Compound Discoverer 3.3 (version 3.3.2.31; Thermo, Waltham). Based on the new peak detection and peak quality scoring algorithm of the software, peak extraction, alignment, correction, and other operations were performed. The unique peak quality rating calculation and filter considerably reduced the interference of background and low-quality peaks. The peaks that were not detected in more than 50% of the QC samples were filtered, the missing values of the undetected peaks were filled based on the Fill Gaps algorithm, and the sum total peak area was normalized. The identification of metabolites was based on self-built library PSNGM Database, mzCloud online library (https://www.mzcloud.org/), LIPID MAPS (https://www.lipidmaps.org/), HMDB (https://hmdb.ca/), MoNA (https://mona.fiehnlab.ucdavis.edu/), and NIST_2020_MSMS spectral library. The MS1mass tolerance was set to 15 ppm, and the MS2 Match Factor Threshold was set to 50.

### Differential Metabolite Analysis

The R package Ropls was employed to separately conduct PCA, PLS-DA, and OPLS-DA for dimensionality reduction of the sample data. Score plots, loading plots, and S-plots were generated to illustrate differences in the metabolic compositions among the samples. Model overfitting was assessed using permutation testing. R^2^X and R^2^Y represent the explained variance of the constructed models for the X and Y matrices, respectively, and Q^2^ indicates the model predictive capability. Higher values approaching 1 indicate a better model fit and accurate classification of training set samples into their original groups. False discovery rate (FDR)-adjusted *P*-value, OPLS-DA-derived variable importance in projection (VIP), and fold change (FC) were calculated to quantify the influence intensity and explanatory power of individual metabolite components on sample classification, facilitating the selection of discriminatory metabolites. Metabolites with FDR-adjusted *P*-value < 0.05, and VIP >1 were considered statistically significant.

Clustering analysis of differential metabolite abundances was performed using the Pheatmap package (version 1.0.12) in R, with heatmaps and trend analysis plots drawn. Venn diagrams and UpSet plots for differentially expressed substances in two-group comparisons were created using VennDiagram (version 1.7.3) and UpSetR (version 1.4.0), respectively. Correlation analysis of differential metabolites was conducted using corrplot (version 4.0.3), and box and violin plots for these metabolites were generated using ggplot2 (version 3.4.1) to display their abundance patterns across different groups. Further machine learning analysis (mlr3verse, version 0.2.7) and ROC curve plotting (version 1.18.2) were applied to the differential metabolites to extract key product information. Functional analysis of differential metabolites primarily involved Kyoto Encyclopedia of Genes and Genomes (KEGG) enrichment analysis using clusterProfiler (version 4.6.0), revealing significantly enriched metabolic pathways and calculating overall differential abundance scores to capture the average and overall trend changes for all differential metabolites within a given pathway, thereby aiding in the identification of critical pathways. The resultant dataset was subjected to downstream analyses using the cloud-based bioinformatics platform of PerSonalbio.

## Results

### QC and Metabolite Identification

Key QC metrics, comprising the number of features retained after filtering, for the positive- and negative-ionization modes are provided in Supplementary Tables S1 and S2, respectively. The overlaid total ion chromatograms in both positive- and negative-ionization modes (Supplementary Fig. S1) showed highly consistent peak response intensity and retention time across all QC samples, indicating excellent instrument stability throughout the analytical process. Metabolite identifications and their corresponding confidence levels are presented in Supplementary Table S3. Representative MS/MS spectra for several key biomarker metabolites are provided in Supplementary Fig. S2 to support their identification.

### PCA and OPLS-DA Analysis

**Fig. 1.**
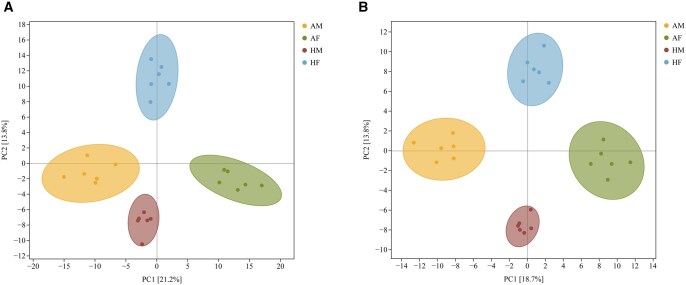
Principal component analysis (PCA) score plot of metabolites from AM, AF, HM, and HF. (A) PCA score plot of metabolites from AM, AF, HM, and HF in the positive ion mode; (B) PCA score plot in the negative ion mode. Each point represents an individual sample with a distinct color/shape combination, denoting a sample group. Ellipses indicate 95% CIs. AF, *Arma chinensis* females; AM, *A. chinensis* males; HF, *Halyomorpha halys* females; HM, *H. halys* males.

**Fig. 2.**
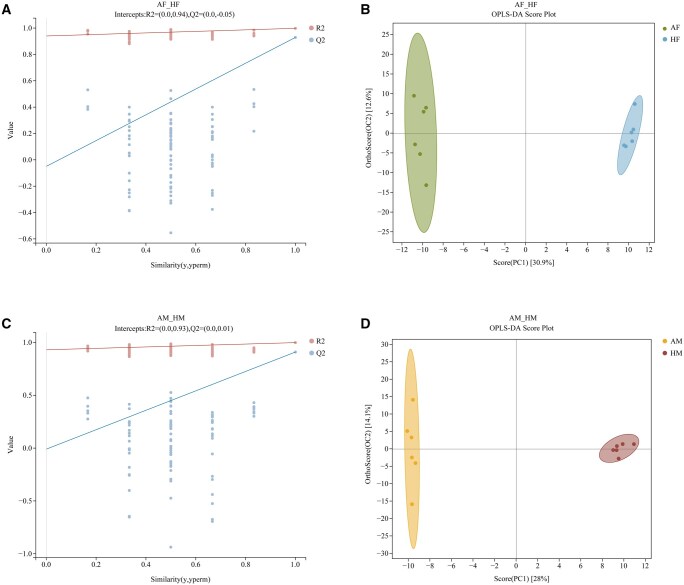
Orthogonal partial least squares-discriminant analysis (OPLS-DA) score plots and permutation validation plots (positive ion mode). (A) OPLS-DA permutation test for AF vs. HF (*R*^2^  *X* = 0.343; *R*^2^  *Y* = 0.998; *Q*^2^ = 0.929); (B) OPLS-DA score plot for AF vs. HF; (C) OPLS-DA permutation test for AM vs. HM (*R*^2^  *X* = 0.42; *R*^2^  *Y* = 0.998; *Q*^2^ = 0.909); and (D) OPLS-DA score plot for AM vs. HM. AF, *Arma chinensis* females; AM, *A. chinensis* males; HF, *Halyomorpha halys* females; HM, *H. halys* males.

**Fig. 3.**
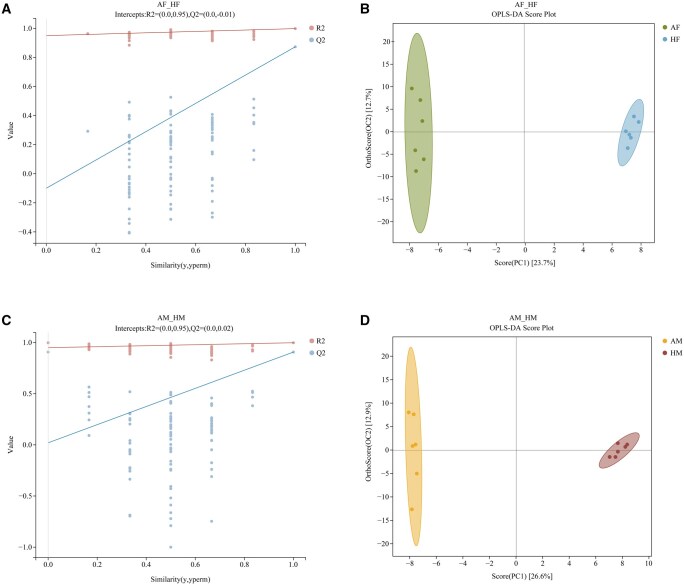
Orthogonal partial least squares-discriminant analysis (OPLS-DA) score plots and permutation validation plots (negative ion mode). (A) OPLS-DA permutation test for AF vs. HF (*R*^2^  *X* = 0.364; *R*^2^  *Y* = 0.998; *Q*^2^ = 0.873); (B) OPLS-DA score plot for AF vs. HF; (C) OPLS-DA permutation test for AM vs. HM (*R*^2^  *X* = 0.395; *R*^2^  *Y* = 0.998; *Q*^2^ = 0.907); and (D) OPLS-DA score plot for AM vs. HM. AF, *Arma chinensis* females; AM, *A. chinensis* males; HF, *Halyomorpha halys* females; HM, *H. halys* males.

To assess and identify differential metabolites, multivariate statistical analyses were applied to the metabolomic data. PCA was performed separately for the AM, AF, HM, and HF metabolite profiles. The PCA score plot (Fig. 1) revealed that all samples clustered within the 95% confidence ellipse, with a distinct separation between *A. chinensis* and *H. halys* samples. However, detailed differences between individual clusters remained unresolved. Subsequently, OPLS-DA, employed to maximize the differences in metabolic profiles between *A. chinensis* and *H. halys*, revealed a pronounced separation of the metabolic profiles (Figs 2B and D and 3B and D). Model parameters confirmed robust modeling and predictive capability, and model validation conformed to established criteria: permutation tests were considered statistically valid if either condition was satisfied, and all permuted *Q*^2^ values were below the original *Q*^2^ value (the rightmost point). The regression line of *Q*^2^ points intersected the vertical axis below zero. The permutation test results (*H. halys* vs. *A. chinensis*, Figs 2A and C and 3A and C) satisfied these validity requirements, supporting the reliability of the model for subsequent analysis. Collectively, these results revealed clear metabolic differences between the 2 insect species, independent of sex.

### Identification and Classification of Differential Metabolites

Based on the OPLS-DA results, metabolites with VIP >1 and statistically significant between-group differences (FDR-adjusted *P*-value < 0.05, *t*-test) were identified as biomarkers, indicating metabolic divergence between *A. chinensis* and *H. halys*. In females (AF vs. HF), 194 differential metabolites were detected (64 upregulated and 130 downregulated in *A. chinensis*), which were categorized into 13 main groups dominated by lipids and lipid-like molecules (16.5%), organic acids and derivatives (14.9%), and organoheterocyclic compounds (14.4%; Table 1). In males (AM vs. HM), 195 differential metabolites were identified (108 upregulated and 87 downregulated in *A. chinensis*), distributed across 12 major categories, primarily comprising organic acids and derivatives (21.5%), lipids and lipid-like molecules (16.4%), and organoheterocyclic compounds (12.3%; Table 2).

**Table 1.** Categories and quantities of differential metabolites between *A. chinensis* and *H. halys* females

Type	Number	Percentage	Up	Down
**Lipids and lipid-like molecules**	32	16.5%	17	15
**Organic acids and derivatives**	29	14.9%	11	18
**Organoheterocyclic compounds**	28	14.4%	4	24
**Benzenoids**	25	12.9%	6	19
**Organic nitrogen compounds**	5	2.6%	3	2
**Alkaloids and derivatives**	4	2.1%	2	2
**Nucleosides, nucleotides, and analogues**	4	2.1%	2	2
**Phenylpropanoids and polyketides**	4	2.1%	1	3
**Organic oxygen compounds**	3	1.5%	0	3
**Homogeneous non-metal compounds**	1	0.5%	0	1
**Organohalogen compounds**	1	0.5%	0	1
**Organometallic compounds**	1	0.5%	1	0
**Other**	57	29.4%	19	38

**Table 2.** Categories and quantities of differential metabolites between *A. chinensis* and *H. halys* males

Type	Number	Percentage (%)	Up	Down
**Organic acids and derivatives**	42	21.5	25	17
**Lipids and lipid-like molecules**	32	16.4	15	17
**Organoheterocyclic compounds**	24	12.3	13	11
**Benzenoids**	21	10.8	16	5
**Phenylpropanoids and polyketides**	7	3.6	5	2
**Organic oxygen compounds**	6	3.1	5	1
**Organic nitrogen compounds**	5	2.6	2	3
**Nucleosides, nucleotides, and analogues**	4	2.1	2	2
**Alkaloids and derivatives**	2	1.0	0	2
**Homogeneous non-metal compounds**	1	0.5	1	0
**Lignans, neolignans, and related compounds**	1	0.5	0	1
**Other**	50	25.6	24	26

**Fig. 4.**
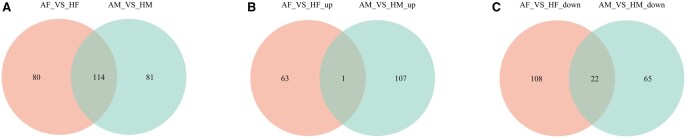
Venn diagrams of overlapping and unique differential metabolites between *Arma chinensis* and *Halyomorpha halys* females and males. (A) All differential metabolites between females (AF vs. HF) and males (AM vs. HM) of both species; (B) all upregulated differential metabolites between females (AF vs. HF) and males (AM vs. HM) of both species; and (C) all downregulated differential metabolites between females (AF vs. HF) and males (AM vs. HM) of both species. AF, *A. chinensis* females; AM, *A. chinensis* males; HF, *H. halys* females; HM, *H. halys* males.

Untargeted metabolomics revealed a high degree of commonality among the differential metabolites between the sexes of *A. chinensis* and *H. halys* (Fig. 4A). In contrast, upregulated metabolites displayed distinct sex- and species-specific patterns, likely reflecting distinct physiological or ecological adaptation mechanisms (Fig. 4B). Twenty-two common downregulated metabolites were detected (Fig. 4C), with greater overlap than in the upregulated metabolites. This implied potential shared regulatory mechanisms in specific metabolic pathways between the 2 species, although significant species-specific differences persisted.

These results collectively highlight shared and unique metabolic features of *A. chinensis* and *H. halys*, offering important insights into their metabolic regulation.

**Fig. 5.**
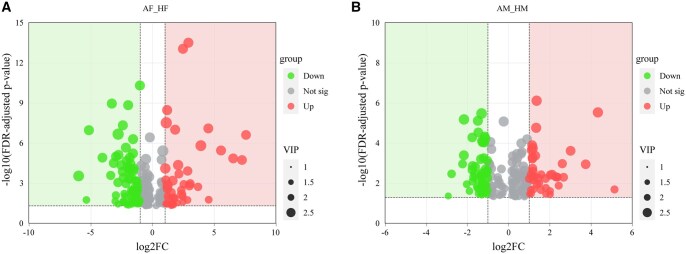
Volcano plots of differential metabolites between female and male *Arma chinensis* and *Halyomorpha halys* adults. (A) Female comparisons (AF vs. HF) and (B) male comparisons (AM vs. HM). *X*-axis: Log_2_(FC) of metabolite abundance between the groups. *Y*-axis: statistical significance of differences (–log_10_ [FDR-adjusted *P*-value]). Red dots: significantly upregulated metabolites (criteria: FDR-adjusted *P*-value < 0.05, |log_2_FC| > 1). Green dots: significantly downregulated metabolites (criteria: FDR-adjusted *P*-value < 0.05, |log_2_FC| > 1). Dot size: variable importance in the projection (VIP) score from multivariate analysis. AF, *A. chinensis* females; AM, *A. chinensis* males; HF, *H. halys* females; HM, *H. halys* males.

Volcano plots revealed significant metabolic divergence between *A. chinensis* and *H. halys* in the sexes. For females (AF vs. HF; Fig. 5), *A. chinensis* exhibited higher levels of lipids and lipid-like molecules, while *H. halys* was enriched in organic acids, their derivatives, and plant-derived secondary metabolites such as alkaloids. A similar pattern of metabolite differences was observed in males (AM vs. HM; Fig. 5B). In both sexes, notable upregulation and downregulation of certain metabolites were observed (FDR-adjusted *P*-value < 0.05, |log_2_FC| > 1).

### Clustering of Differential Metabolites and KEGG Enrichment Analysis

**Fig. 6.**
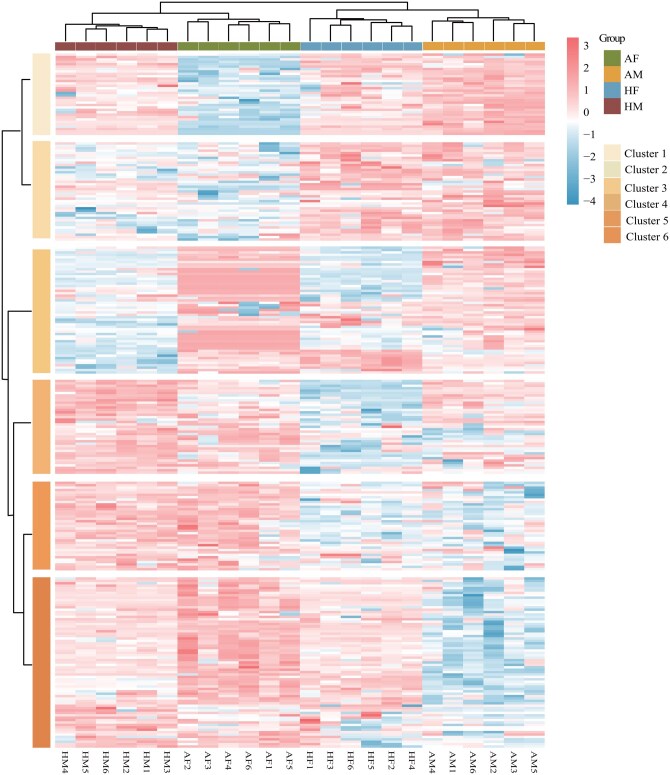
Hierarchical clustering heatmap of differential metabolites between *Arma chinensis* and *Halyomorpha halys*. The top color bars indicate the sample groups (AF, AM, HF, and HM). Color gradient: blue (low expression) and red (high expression) intensity represent *z*-score normalized metabolite abundance. AF, *A. chinensis* females; AM, *A. chinensis* males; HF, *H. halys* females; HM, *H. halys* males.

To clarify the relationship between differential metabolites and nutritional metabolism in *A. chinensis* and *H. halys*, a hierarchical clustering heatmap was constructed, and KEGG enrichment analysis was performed on the identified differential metabolites. The heatmap revealed distinct clustering of metabolic profiles between *A. chinensis* and *H. halys* (Fig. 6). Analysis of their expression patterns identified metabolites with marked differences between the 2 species (Supplementary Table S4).

**Fig. 7.**
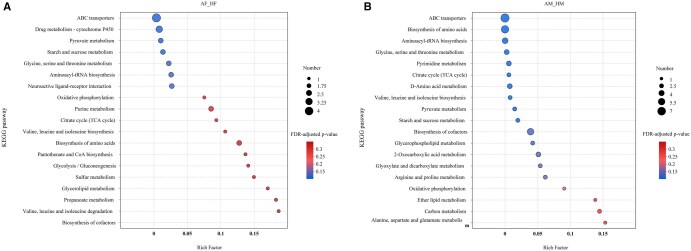
The Kyoto encyclopedia of genes and genomes (KEGG) enrichment analysis of differential metabolites in *Arma chinensis* and *Halyomorpha halys.* (A) Enrichment bubble plot for female comparisons (AF vs. HF); and (B) enrichment bubble plot for male comparisons (AM vs. HM). Bubble color indicates the significance level (–log_10_ [FDR-adjusted *P*-value]); bubble size represents the enrichment factor. AF, *A. chinensis* females; AM, *A. chinensis* males; HF, *H. halys* females; HM, *H. halys* males.

KEGG enrichment analysis of 194 differential metabolites between female *A. chinensis* and *H. halys* (AF vs. HF) revealed significant enrichment in 33 pathways involving 60 metabolites (Supplementary Table S5). Carbohydrate metabolism showed the highest degree of enrichment (9 pathways, 11 metabolites), followed by amino acid metabolism (4 pathways), while energy metabolism, nucleotide metabolism, and lipid metabolism each contained 2 enriched pathways. The bubble plot in Fig. 7A clearly demonstrates that the ATP-binding cassette (ABC) transporter pathway was the most significantly enriched, followed by pyruvate, starch, and sucrose metabolism.

For comparisons between males (AM vs. HM), analysis of 195 differential metabolites demonstrated enrichment in 36 pathways, comprising 90 metabolites (Supplementary Table S6). Similarly, carbohydrate metabolism pathways were the most enriched (9 pathways, 13 metabolites), and amino acid metabolism pathways were notably increased (8 pathways). Three pathways were enriched for cofactors/vitamins and lipid metabolism. ABC transporters remained the most significantly enriched pathway in males, followed by amino acid and aminoacyl-tRNA biosynthesis (Fig. 7B).

The integrated analysis revealed that both sexes had significant differences in metabolites enriched in ABC transporter pathways, amino acid metabolism, and carbohydrate metabolism. Among these, the ABC transporter pathway was most significantly enriched in the comparisons between females (AF vs. HF) and males (AM vs. HM). This suggested potential differences between *A. chinensis* and *H. halys* in functions such as nutrient absorption and toxin efflux (Bariami et al. 2012, Dermauw and Van Leeuwen 2014, Gott et al. 2017). Specifically, *A. chinensis* exhibited marked enrichment in lipids and lipid-like molecules, aligning with the high-energy demands of its predatory behavior. In contrast, *H. halys* accumulated plant-derived secondary metabolites and detoxification-related compounds, reflecting its adaptive evolution in response to plant defense chemicals, along with the activation of xenobiotic degradation pathways that further support its evolved adaptive strategy against phytochemical defenses (Amezian et al. 2021).

These metabolic signatures distinctly reflect the divergent dietary niches of the species: enhanced lipid metabolism supporting the predatory behavior of *A. chinensis* and robust detoxification of plant allelochemicals and utilization of plant metabolites characterizing the herbivorous adaptation of *H. halys*.

## Discussion

This study employed untargeted metabolomics to systematically compare the global metabolic profiles of 2 closely related species: the predatory stink bug, *A. chinensis* (Fallou), and the phytophagous stink bug, *H. halys* (Stål). The analysis revealed significant interspecific metabolic differences. By integrating multivariate statistics with pathway enrichment results, the observed metabolic patterns were found to be consistent with known physiological distinctions between predatory and phytophagous lifestyles, particularly in energy metabolism and nutrient allocation. At the molecular level, these patterns point to specific metabolic features that correlate with the divergent trophic niches of these species. This work provides empirical metabolomic data that contribute to the understanding of insect dietary ecology and illustrates the potential of metabolomics for investigating phenotypic variations within an ecological framework. Although the use of 6 biological replicates per group is a common and accepted practice in similar untargeted metabolomic comparison studies within this field, we acknowledge that for high-dimensional data, a larger sample size would provide greater statistical power. The key findings of this study are based on multivariate statistical analysis (OPLS-DA) and the screening of differential metabolites with stringent FDR correction—methods that, to some extent, account for the multidimensional nature of the data. However, a larger sample size in future research would facilitate the detection of differential metabolites with smaller effect sizes.

The diversification of insect feeding habits, such as phytophagy and predation, is a key aspect of insect ecological diversity, with dietary differences being a significant factor associated with insect evolution. Molecular evidence indicates that the expansion of detoxification enzyme systems often correlates with varied feeding habits. For instance, notable differences in the UDP-glycosyltransferase (UGT) gene family have been observed between polyphagous and oligophagous *Spodoptera* species. Polyphagous insects, such as *S. frugiperda* and *Spodoptera exigua*, possess expanded UGT gene families, which may assist in metabolizing diverse plant secondary metabolites, such as 2,4-dihydroxy-7-methoxy-1,4-benzoxazin-3-one and gossypol, potentially facilitating the use of a wider range of host plants. In contrast, loss-of-function mutations in UGT genes apparently restrict oligophagous species, such as *Spodoptera picta*, in metabolizing certain plant toxins, likely limiting their dietary breadth (Wang et al. 2024a). Similarly, the expansion of UGT genes in phytophagous ladybird beetles may support the metabolism of plant toxins, whereas the expansion of odorant-binding protein genes in predatory ladybirds could enhance the recognition of prey chemical signals. These gene family dynamics suggest molecular pathways that may facilitate insect responses to environmental pressures linked to different feeding strategies (Huang et al. 2025). Additionally, in response to insecticide exposure, *A. chinensis* can rapidly metabolize toxins and restore activity by upregulating detoxification genes, including cytochrome P450 monooxygenases, carboxylesterases, and ABC transporters, indicating metabolic flexibility that supports survival in pesticide-contaminated environments while maintaining predatory function (Wang et al. 2024c).

Distinct feeding habits are strongly associated with differences in the gut microbial communities of insects. Phytophagous insects, such as the migratory locust (*Locusta migratoria* L.) and the silkworm (*B. mori* L.), harbor gut microbiomes enriched with bacterial taxa involved in the degradation of plant cellulose and toxins. For instance, *L. migratoria* shows an increased abundance of Proteobacteria under intensive grazing conditions, which contributes to coping with fluctuating plant resources (Shen et al. 2025). Similarly, *Pseudomonas fulva* in the gut of *B. mori* is implicated in the breakdown of mulberry-derived alkaloids, potentially assisting in host detoxification (Zhang et al. 2024a). In contrast, predatory insects, such as the multicolored Asian lady beetle (*Harmonia axyridis*, Pallas), possess gut microbiota that closely resembles that of their prey. Furthermore, the gut microbiota of adults appears more responsive to dietary shifts than that of larvae (Zhang et al. 2024b). Previous research on *A. chinensis* and *H. halys* revealed clear differences in microbiomes linked to feeding, digestion, and reproduction (Cheng et al. 2025); the metabolic differences identified in the present study are consistent with the earlier findings.

*Arma chinensis* relies on high protein and lipid resources obtained from its prey. The enriched metabolites, such as glycerophospholipids and fatty acids, could serve as efficient energy reserves, potentially supporting active hunting and rapid movement. Lipids also appear to contribute to defense systems, which might enhance the capacity of the organism to respond to prey resistance or environmental stress (Downer and Matthews 1976, Klowden 2008, Yew and Chung 2015, Wrońska et al. 2023). In contrast, the metabolic profile of *H. halys* reflects metabolic strategies commonly observed in phytophagous insects, which are often associated with countering host plant chemical defenses. For example, *H. halys* may mitigate the effects of plant toxins by accumulating alkaloids and phenolics (Hartmann and Ober 2008, Trigo 2011, War et al. 2019) or by efficiently converting energy from nutrient-poor plant sap through organic acid derivatives (Li et al. 2002, Francis et al. 2005, Miyagi et al. 2013). These pronounced metabolic differences highlight a strong association between feeding habits and the physiological profiles of insects, even among closely related species.

Metabolic differences between female and male individuals reflect a close linkage between reproductive and ecological functions. Lipid reserves are important for reproductive success (Hou et al. 2017), as insects typically require substantial lipids for vitellogenin synthesis during egg production. This pattern aligns with the higher proportion of upregulated lipids in AF than in AM. In contrast, the more active protein synthesis-related metabolism observed in males may correspond to their potentially greater need for mobility and hunting to secure mating resources. Such sexual dimorphism in nutritional profiles has also been documented for other insects. For example, female larvae of the yellow fever mosquito, *Aedes aegypti* L., require more bacterially derived B vitamins, such as biotin, than males and are more tolerant of elevated biotin concentrations, a pattern that could relate to the metabolic demands of reproduction (Romoli et al. 2024). Similarly, in *B. mori*, the estrogen-related receptor is highly expressed in the midgut and fat body, with its regulation of sugar, lipid, and trehalose transport more pronounced in females, consistent with their increased energy demands during embryonic development and silk protein synthesis (Hou et al. 2025).

In summary, untargeted metabolomics revealed significant metabolic differences between the predatory stink bug *A. chinensis* (Fallou) and the phytophagous stink bug *H. halys* (Stål), corresponding to their contrasting dietary ecologies. *Arma chinensis* was enriched in lipids and lipid-like molecules, whereas *H. halys* showed a predominance of organic acid derivatives and plant-derived secondary metabolites. Sexual dimorphism in metabolism was also observed, with lipid enrichment in females (AF/HF) suggestive of energy reserves for oogenesis, whereas males (AM/HM) exhibited metabolic profiles biased toward protein synthesis, which may relate to their distinct behavioral demands. The KEGG pathway analysis revealed significant enrichment in ABC transporter pathways, carbohydrate metabolism, and amino acid metabolism, pointing to differences in core metabolic networks linked to feeding habits. Hence, this study identifies key metabolic differences and pathways associated with dietary divergence in pentatomids and contributes to the understanding of insect physiological ecology. The findings may also inform the development of pest management strategies.

This study had some inherent limitations that must be addressed in future research. First, because of the use of whole-body extracts for untargeted metabolomics, the identified metabolic profiles represent integrated signals from host tissues, hemolymph, gut contents, and associated microbiota. Consequently, the specific tissue or cellular origin of the differential metabolites is uncertain, which complicates the interpretation of their biological context and functional relevance. Second, as an exploratory analysis, these findings require orthogonal validation. It should be noted that all metabolite identifications in this study were based on high-resolution mass spectrometry and MS/MS spectral library matching (MSI Level 2), and confirmation using commercial reference standards was not performed. Based on the significance of differences in relative abundance (ie the absolute value of log_2_FC) of metabolites between *A. chinensis* and *H. halys*, MS2 spectra of 4 metabolites were selected for reference (Supplementary Fig. S2). Therefore, future targeted studies should focus on the absolute quantification and verification of these candidate biomarkers. Subsequent investigations should employ tissue-specific dissections—such as isolation of the midgut, fat body, or hemolymph—to more precisely localize metabolic variations. Used in conjunction with targeted mass spectrometry techniques for the absolute quantification of key candidate metabolites, these approaches will help further elucidate their biological roles and mechanistic contributions. Addressing these limitations in follow-up studies will strengthen the conclusions drawn from the current exploratory dataset.

## Supplementary Material

ieag005_Supplementary_Data

## Data Availability

The metabolomics dataset generated in this study has been deposited in the Metabolomics Workbench repository (Study ID: ST004454). The data are publicly accessible via the project DOI: http://dx.doi.org/10.21228/M8XG1G.
