# Supplementary material for: Untargeted metabolomics reveals divergent metabolic profiles between the predatory Arma chinensis and the Phytophagous Halyomorpha halys
Source: J Insect Sci. 2026 Feb 2;26(1):ieag005. doi: 10.1093/jisesa/ieag005 (PMC12863074; doi:10.1093/jisesa/ieag005)
Supplement: ieag005_Supplementary_Data [file ieag005_supplementary_data.zip › Supplementary Figure S1.docx]

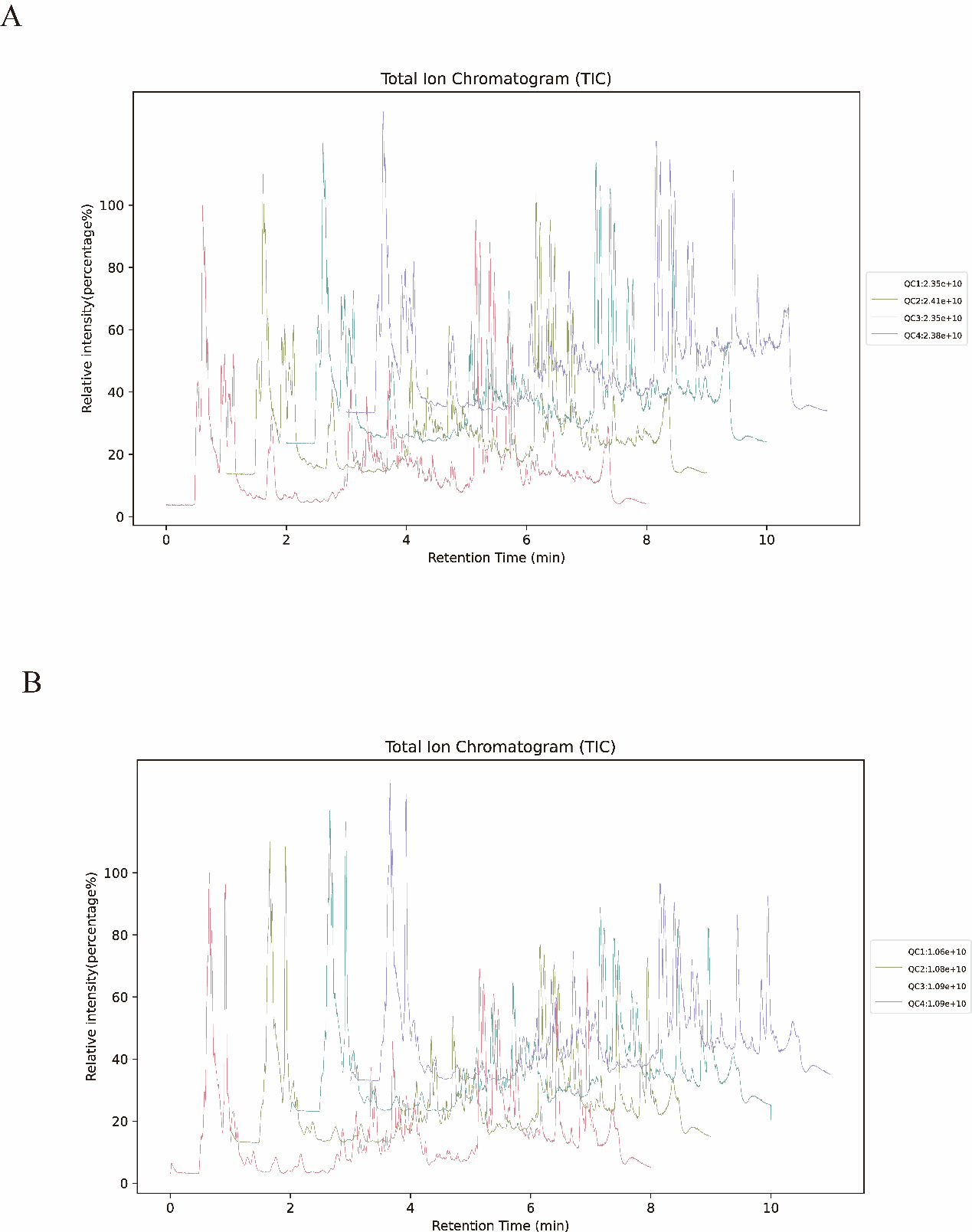


Figure S1: Overlaid total ion chromatograms (TICs) in positive- and negative-ionization modes. (A)The combined total ion chromatogram (TIC) in the positive ionization mode. (B)The combined total ion chromatogram (TIC) in the negative ionization mode.
