# Supplementary material for: Untargeted metabolomics reveals divergent metabolic profiles between the predatory Arma chinensis and the Phytophagous Halyomorpha halys
Source: J Insect Sci. 2026 Feb 2;26(1):ieag005. doi: 10.1093/jisesa/ieag005 (PMC12863074; doi:10.1093/jisesa/ieag005)
Supplement: ieag005_Supplementary_Data [file ieag005_supplementary_data.zip › Supplementary Figure S2.docx]

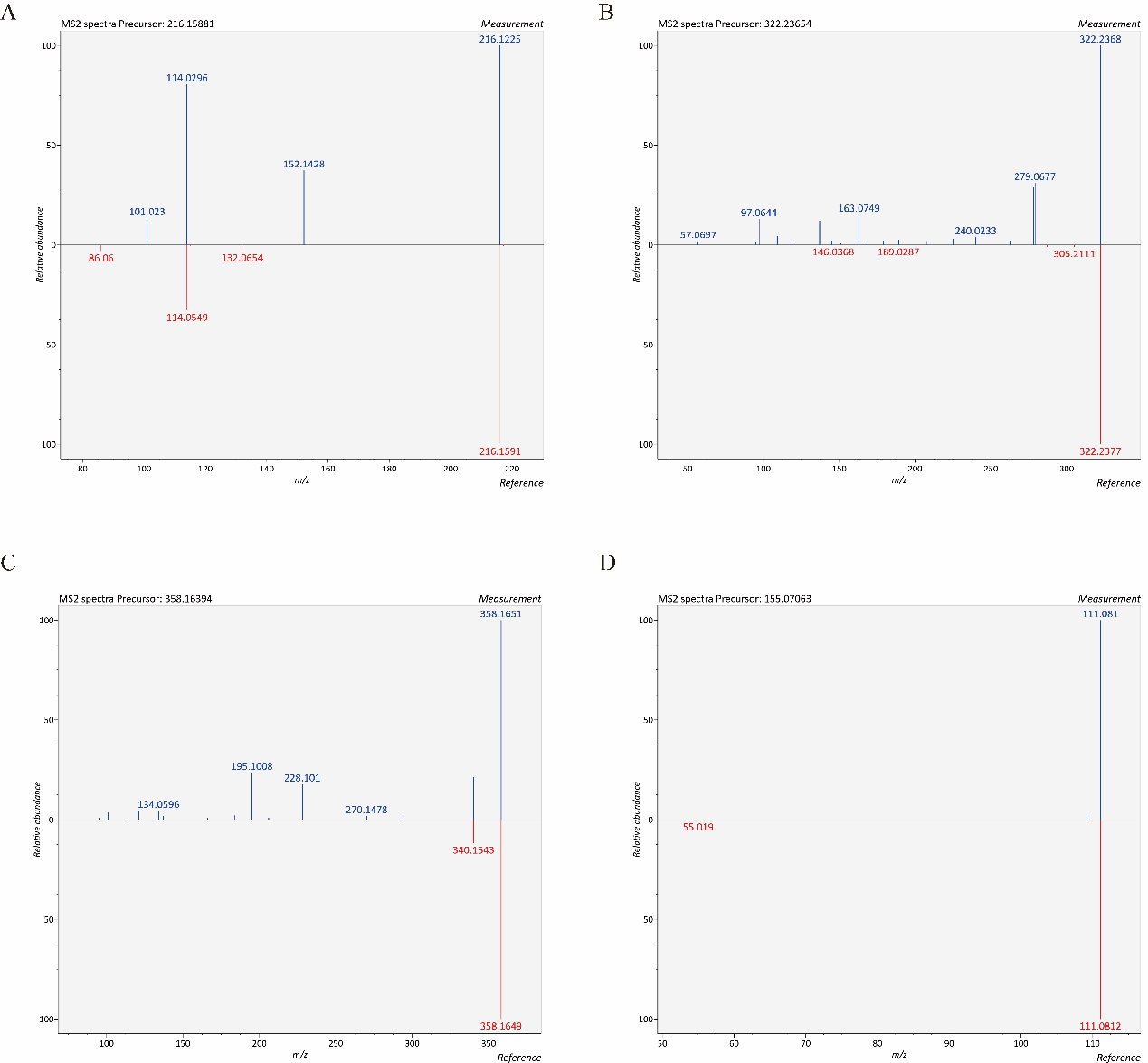
Figure S2: Representative MS/MS spectra of key biomarker metabolites.

(A) The MS2 spectrum of hexaminolevulinate. (B) The MS2 spectrum of 16-hydroxydehydroepiandrosterone. (C) The MS2 spectrum of 2,3-Dihydroxy-9,10,11-trimethoxy-5,8,13,13a-tetrahydroxy-6H-dibenzo(a,g)chinolysin. (D) The MS2 spectrum of 2-Propylglutaric acid.
